# Bladder tumor ILC1s undergo Th17‐like differentiation in human bladder cancer

**DOI:** 10.1002/cam4.4243

**Published:** 2021-09-08

**Authors:** Neelam Mukherjee, Niannian Ji, Xi Tan, Chun‐Lin Lin, Emily Rios, Chun‐Liang Chen, Tim Huang, Robert S. Svatek

**Affiliations:** ^1^ Department of Urology University of Texas Health San Antonio (UTHSA) San Antonio Texas USA; ^2^ Department of Molecular Medicine University of Texas Health San Antonio (UTHSA) San Antonio Texas USA

**Keywords:** bladder cancer, clustering, ILC1s, innate lymphoid cells, Th17

## Abstract

**Purpose:**

Human innate lymphoid cells (hILCs) are lineage‐negative immune cells that do not express rearranged adaptive antigen receptors. Natural killer (NK) cells are hILCs that contribute to cancer defense. The role of non‐NK hILCs in cancer is unclear. Our study aimed to characterize non‐NK hILCs in bladder cancer.

**Experimental design:**

Mass cytometry was used to characterize intratumoral non‐NK hILCs based on 35 parameters, including receptors, cytokines, and transcription factors from 21 muscle‐invasive bladder tumors. Model‐based clustering was performed on t‐distributed stochastic neighbor embedding (t‐SNE) coordinates of hILCs, and the association of hILCs with tumor stage was analyzed.

**Results:**

Most frequent among intratumoral non‐NK hILCs were hILC1s, which were increased in higher compared with lower stage tumors. Intratumoral hILC1s were marked by Th17‐like phenotype with high RORγt, IL‐17, and IL‐22 compared to Th1 differentiation markers, including Tbet, perforin, and IFN‐γ. Compared with intratumoral hILC2s and hILC3s, hILC1s also had lower expression of activation markers (NKp30, NKp46, and CD69) and increased expression of exhaustion molecules (PD‐1 and Tim3). Unsupervised clustering identified nine clusters of bladder hILCs, which were not defined by the primary hILC subtypes 1–3. hILC1s featured in all the nine clusters indicating that intratumoral hILC1s displayed the highest phenotypic heterogeneity among all hILCs.

**Conclusions:**

hILC1s are increased in higher stage tumors among patients with muscle‐invasive bladder cancer. These intratumoral hILC1s exhibit an exhausted phenotype and Th17‐like differentiation, identifying them as potential targets for immunotherapy.

## Graphical abstract

This study characterizes the diversity of tumor‐infiltrating ILCs within muscle‐invasive bladder cancer. Unique ILC subtypes were identified that differed from traditional classifications of ILCs. We further found that bladder ILC1s displayed a Th17‐like phenotype and correlated with tumor stage, suggesting further investigations into their role in cancer and potential immunotherapy targets.

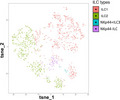

## 1 INTRODUCTION

Innate lymphoid cells (ILCs) are components of the innate immune system that do not express somatically generated adaptive antigen receptors. They are identified as CD45^+^ cells that lack major lineage markers (Lin^−^), including markers of B cells (CD19), T cells (CD3), and dendritic cells/macrophages (CD14, ILT3).^1^, ^2^, ^3^, ^4^ ILC groups represent innate counterparts to distinct T‐cell subsets: Natural Killer (NK) cells mirror CD8^+^ cytolytic T cells and ILC groups 1, 2, and 3 mirror T helper (Th) subsets Th1, Th2, and Th17, respectively, according to their cytokine and transcription factor production.^5^, ^6^ Thus, group 1 ILCs (ILC1s) are regulated by the transcription factor Tbet and secrete IFN‐γ, granzyme B, and perforin^7^; group 2 ILCs (ILC2s) are regulated by GATA‐3 and produce IL‐4, IL‐9, IL‐5, and IL‐13^8^; and group 3 ILCs (ILC3s) are regulated by retinoic acid receptor‐related orphan receptor γ‐t (RORγt) and produce IL‐17 and IL‐22.^9^ While useful for classification, this straightforward nomenclature of ILCs is evolving based on an increased understanding of ILC biology, including their functional plasticity.^10^

ILCs accumulate at pathogen entry sites, including mucosal surfaces and skin. Thus, ILCs were immediately recognized for their importance in fighting infections.^11^, ^12^ Subsequent work identified contributions of ILCs to cancer progression, including both pro‐ and anti‐tumor effects depending on the tumor environment.^13^, ^14^, ^15^, ^16^ While a role for NK cell human ILCs (hILCs) in regulating the growth of human tumors is established, the importance of non‐NK hILCs in cancer is less defined.

Urinary bladder cancer is broadly divided into two major types based on tumor stage. Non‐muscle‐invasive bladder tumors are treated with endoscopic surgical removal and/or intravesical chemotherapy or Bacillus Calmette–Guérin (BCG).^17^ Muscle‐invasive bladder tumors, which have not metastasized, are typically treated with systemic chemotherapy and radical cystectomy.^18^ In muscle‐invasive tumors, a CD56^bright^ cytotoxic subset of NK hILCs was associated with improved patient survival.^19^ However, the role of non‐NK hILCs in bladder cancer is unclear. A small but measurable quantity of non‐NK hILCs was observed in the urine of patients with non‐muscle‐invasive bladder cancer who were treated with intravesical BCG, including group 2 ILCs and, to a lesser extent, groups 1 and 3 ILCs.^20^ Urine ILC2s were also negatively correlated with BCG response in bladder cancer and their frequency was linked to the recruitment of suppressive myeloid cells, suggesting ILCs play important roles in bladder cancer and bladder ILCs skew type 2 immune responses and suppress immune function despite their small absolute numbers.^20^ However, to the best of our knowledge, no study has investigated the frequency, diversity, and phenotype of bladder tumor‐infiltrating non‐NK hILCs. Our study for the first time characterizes the bladder tumor ILCs in bladder cancer patients.

We hypothesized that non‐NK ILCs represent an infrequent but important population in bladder cancer progression. We sought to characterize the diversity of non‐NK hILCs in muscle‐invasive bladder tumors using cystectomy specimens, which provide large amounts of tissue necessary to identify rare immune cell populations. Mass cytometry by time‐of‐flight (CyTOF) was applied to improve upon spectral limitations of flow cytometry, providing a comprehensive characterization of bladder intratumoral hILCs, including simultaneous activation status and effector cytokine and transcription factor expression.

## 2 MATERIALS AND METHODS

### 2.1 Bladder cancer patient cohort

Patients were recruited through a local Institutional Review Board (IRB)‐approved observational cohort study, which collected clinical data and bladder tissue for analysis (IRB # BCR20120159H). Eligible patients were 18 years of age or older and had a confirmed or suspected diagnosis of bladder cancer. All patients provided written informed consent. Patient demographics, pathology and imaging reports, physical exam and laboratory assessments, and specimen tracking data were entered prospectively into a secured web‐based REDCap database system. This study's involvement with human subjects complies with the Declaration of Helsinki.

### 2.2 **Preparation of single‐cell suspension** **from human bladder tumor specimens**

Bladder tumors were surgically excised under sterile conditions as per standard of care. A portion of the tumor was separated and placed in Roswell Park Memorial Institute (RPMI) 1640 medium containing 1% antibiotic (penicillin–streptomycin) and transported on ice. Fresh tumor tissues were washed with phosphate‐buffered saline (PBS) and minced into 1–2 mm pieces and incubated in digestion solution (1 mg/ml collagenase IV, 0.25% trypsin, and 0.25 mg/ml DNAse I) for 40 min at 37°C, 5% CO_2_. After digestion, the enzymes are neutralized by the addition of complete RPMI containing 10% fetal bovine serum (FBS), and the samples were filtered through 100 µM cell strainer to produce single cell suspensions. Single cell suspensions were cryopreserved and stored at −150°C until analyzed. Cystectomy specimens were analyzed because of the large volume of tissue available following bladder removal.

### 2.3 CyTOF staining

CyTOF staining was conducted using single‐cell suspensions derived from bladder tumor specimens (*n* = 21 patients with muscle‐invasive [≥T2] urothelial carcinoma of the bladder). To define the phenotypic diversity of human bladder tumor innate lymphoid cells, we designed a CyTOF panel of 36 antibodies. Cells were thawed in Hank's Balanced Salt Solution without Ca^2+^ or Mg^2+^+10% FBS and the number of viable cells was quantified using trypan blue. Prior to surface staining, cells were stained with cisplatin for discrimination of dead cells from live cells. Cells were then stained with the cell surface antibody cocktail containing: anti‐human CD45, PD‐1, Tim3 CD94, CD56, TIGIT, CD103, CD314 (NKG2D) CD127 (IL‐7Ra), ILT3, CD196 (CCR6), CD294 (CRTH2), CD335 (NKp46), CD161, CD337 (NKp30), CD69, CD38, CD57, CD226, CD16, CD117 (c‐Kit), CD14, CD19, CD8a, CD3, NKp44, and CD4 for 30 min (see Table S1 for clone list and metal). After washing, cells were fixed, permeabilized with MaxPerm‐S buffer for 30 min before staining with the intracellular antibody cocktail containing: anti‐human RORγt, Eomes, IFN‐γ, IL‐13, IL‐22, IL‐17A, perforin, GATA‐3, and Tbet for 30 min. After washing steps, cells were stained for Cell‐ID Intercalator‐Ir to discriminate single nucleated cells from doublets. Finally, cells were resuspended in Cell Acquisition Solution (CAS)‐bead solution to 1 million cells/ml before acquisition of data on Helios.

#### Antibody conjugation

Purified antibodies lacking carrier proteins were conjugated using the Maxpar labeling kit and according to the protocol provided by Fluidigm.

### 2.4 CyTOF data analysis

CyTOF data in FCS (Flow Cytometry Standard) format were first gated in Cytobank (www.cytobank.org) for single CD45^+^ cells. Then 5000 CD45^+^ cells sampled from each tissue sample (total *n* = 105,000) were merged and autoLgcl transformed with R package *cytofkit*. ILCs were then identified as CD3^−^, CD14^−^, CD19^−^, ILT3^−^, CD56^−^, and CD127^+^ cells, where expression levels ≥1 as positive (+) and <1 as negative (−). These 1500 ILCs were subsequently categorized into ILC1/2/3 based on their expression of CRTH2, c‐Kit, and NKp44. Tumor‐infiltrating ILCs were designated based on phenotypical marker profiles as follows: non‐NK ILC1s were identified as CD45^+^Lineage (CD3, CD19, CD14, ILT3)^−^CD56^−^CD127^+^CRTH2^−^c‐Kit^−^; ILC2s defined as CD45^+^Lin^−^CD56^−^CD127^+^CRTH2^+^; and ILC3s defined as CD45^+^Lin‐CD56‐CD127^+^CRTH2^−^c‐Kit^+^, which were further subdivided into NKp44^+^ILC3s and NKp44^−^ILC3s.^20^, ^21^, ^22^ Though NK cells are sometimes included in group 1 ILCs and remain the most researched member of the ILC family,^19^, ^23^, ^24^ here we examine non‐NK ILCs.

### 2.5 Statistics

A script (R) was written to filter out target cells and ILC 1/2/3 were identified. In the script, expression levels less than 1 are considered negative, and higher or equal to 1 are considered positive. With this criterion, in all CD45^+^ cells, the percentages of ILC1/2/3 are calculated. The association of ILCs with tumor stage was assessed using an unpaired *t*‐test. t‐distributed stochastic neighbor embedding (t‐SNE) was conducted on the expression of these 10 markers of ILCs and plotted with R package *ggplot2*. Model‐based clustering was performed on the t‐SNE coordinates of ILCs with R package *mclust*. Violin plots were made with R package *ggplot2*. Duncan's multiple range test was used to compare the expression of individual markers among different groups with the *PostHocTest* function in the R package *DescTools*.

## 3 RESULTS

### 3.1 Bladder tumor‐infiltrating hILC1s are associated with increased bladder tumor stage

**FIGURE 1:**
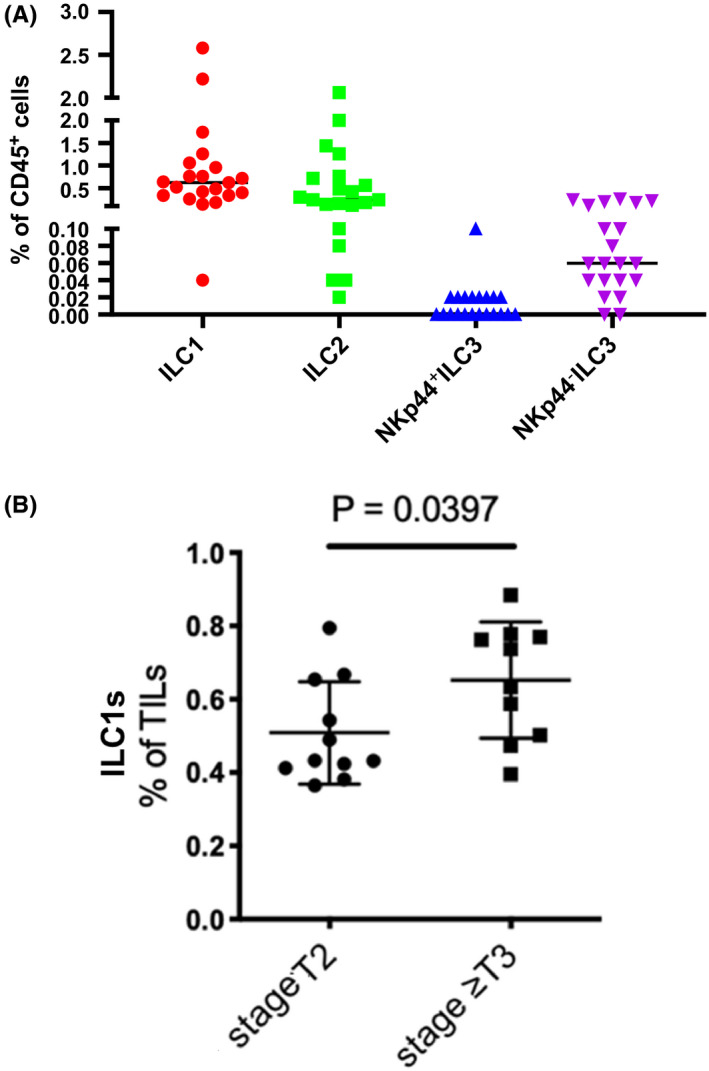
ILC1s are the most abundant tumor‐infiltrating innate lymphoid cells in bladder cancer and are associated with higher bladder tumor stage. Human bladder tumor tissues (*n* = 21) were harvested and processed into single cell suspensions and analyzed with CyTOF. (A) Non‐NK ILC1s were identified as CD45^+^Lineage (CD3, CD19, CD14, ILT3)^−^CD56^−^CD127^+^CRTH2^−^c‐Kit^−^; ILC2s defined as CD45^+^Lin^−^CD56^−^CD127^+^CRTH2^+^; and ILC3s defined as CD45^+^Lin‐CD56‐CD127^+^CRTH2^−^c‐Kit^+^, which were further subdivided into NKp44^+^ILC3s and NKp44^−^ILC3s. A script (R) was written to filter out target cells and ILC 1/2/3 were identified. In the script, expression levels less than 1 are considered negative, and higher or equal to 1 are considered positive. With this criterion, in all CD45^+^ cells, the percentages of ILC1/2/3 are calculated. (B) % of ILC1s is plotted across the pathological stage in bladder cancer

Clinical and pathologic characteristics of the patients are depicted in Table 1. The majority of the patients were male (80.95%). The age ranged between 66.71 and 77.01 years. Pathologic stage was T2 in 12 (57.14%), T3 in 6 (28.57%), and T4 in 3 (14.29%) patients. The most frequent non‐NK hILCs among intratumoral hILCs were group 1 ILCs comprising of 0.78% of CD45^+^ tumor‐infiltrating lymphocytes (TILs), followed by group 2 ILCs (0.54% of TILs), hNKp44^+^ILC3s (0.1% of TILs), and hNKp44^−^ ILC3s (0.09% of TILs) (Figure 1A). Groups 2 and 3 ILCs were not associated with the tumor stage. Given that published data on non‐NK ILC1s demonstrated contributions to antitumor responses including direct cytotoxicity,^13^ production of IFN‐γ and granzyme B,^13^, ^25^, ^26^ we expected non‐NK hILC1s to be associated with decreased disease stage. Surprisingly, however, patients with ≥T3 tumors had a significantly higher percentage of hILC1s compared to patients with T2 tumors (Figure 1B), suggesting that bladder intratumoral non‐NK hILC1s could be tumor‐promoting or exhibit functional plasticity as a result of the bladder tumor environment.

**TABLE 1:** Characteristics of patient cohort. The clinical parameters of the selected bladder patient cohort (*n* = 21) are listed

Mean (range) age	71.86 (66.71–77.01)
Gender	
Female	4 (19.05%)
Male	17 (80.95%)
Ethnicity	
Hispanic	6 (28.57%)
Non‐Hispanic	15 (71.43%)
Stage	
T2	12 (57.14%)
T3	6 (28.57%)
T4	3 (14.29%)
Histologic subtype	
Pure urothelial carcinoma	13 (61.90%)
Urothelial carcinoma with squamous differentiation	7 (33.33%)
Urothelial carcinoma with small cell carcinoma	1 (4.76%)
Prior chemotherapy	
Yes	8 (38.10%)
No	13 (61.90%)
Carboplatin	1 (4.76%)
Gemcitabine	1 (4.76%)
Gemzar/Cisplatin	4 (19.05%)
Gemcitabine/Taxol	1 (4.76%)
Unknown	1 (4.76%)
Prior radiation	
Yes	3 (14.29%)
No	18 (85.71%)
Any tobacco use	
Yes	16 (76.19%)
No	5 (23.81%)

### 3.2 Bladder tumor hILC1s display Th17‐like differentiation

**FIGURE 2:**
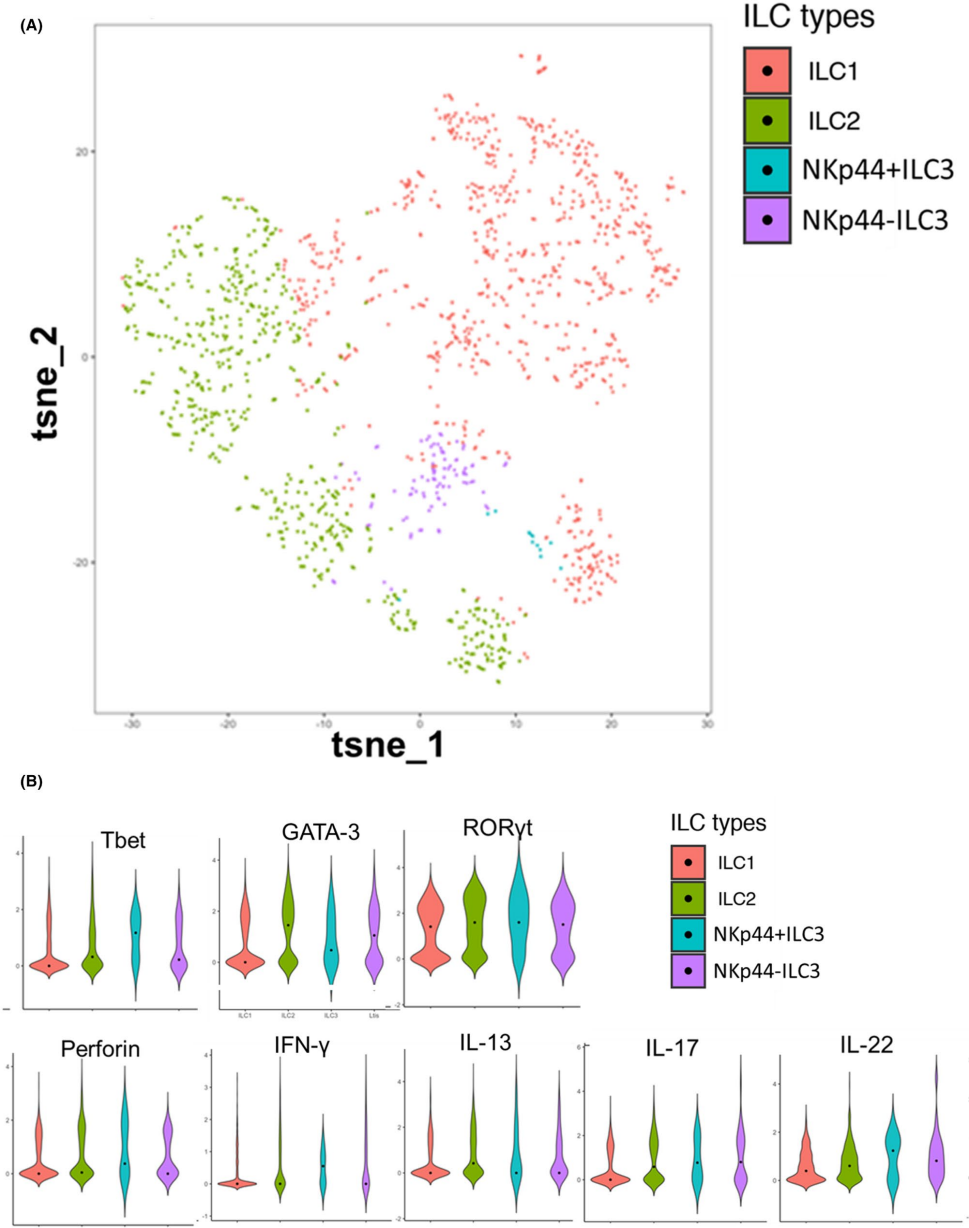
Different ILCs exhibit differential expression of cytokine and regulatory markers. (A) A t‐SNE plot showing the expression of different markers used in the identification of ILCs. (B) Different expressions of transcription factors and cytokines in ILC1, ILC2, and ILC3s are plotted as violin plots

We next examined the phenotypic diversity of bladder hILCs. ILC function is tightly regulated by activating and inhibitory receptors and the combinatorial expression of these molecules characterizes the diversity of hILCs. t‐SNE dimensionality reduction was used to provide an overview of the reconstituted bladder intratumoral hILCs (Figure 2A). Violin plots and bar graphs were used to visualize the scaled expression of differentially expressed Th cytokines and transcription factors across bladder tumor hILCs (Figure 2B; Figure S1). Type 1 cytokine IFN‐γ which is generally expressed by hILC1s, was significantly lower in bladder hILC1s compared with hILC2s (*p* < 0.001, Figure 2; Table S2). In addition, Tbet, a type 1 transcription factor, was lower in bladder hILC1s compared with bladder hILC2s (*p* < 0.001; Figure 2B; Table S2). As expected, hILC2s expressed higher levels of GATA‐3 compared to Tbet and IL‐13 compared to IFN‐γ consistent with a Th2 differentiation profile. Surprisingly, Th17‐like differentiation, including RORγt, IL‐17, and IL‐22 were highly expressed in hILC1s. Compared with IFN‐γ, IL‐17 and IL‐22 were significantly higher in hILC1s (*p* < 0.0001). Furthermore, Tbet was significantly lower compared with RORγt in hILC1 cells (*p* < 0.0001). These data support a Th17 over a Th1 differentiation profile in bladder tumor hILC1s.

Next, we compared the expression of activation or cytotoxicity receptors (NKp30 and NKp46), activation marker (CD69), and exhaustion markers (PD‐1, Tim3, and CD38) among the hILC subsets. Bladder tumor hILC1s had lower expression of NKp30, NKp46, and CD69 compared with hILC2s (*p* < 0.04), **(**Figure S2; Table S3 **)** indicating a reduced cytotoxic capacity of hILC1s compared with hILC2s. hILC2s, on the other hand, had the highest expression of activation receptors and exhaustion markers compared with other hILCs, suggesting that these cells were more chronically activated in bladder tumors compared with other hILCs (*p* < 0.04) (Figure S2; Table S3). CD103 and CD69 were highly co‐expressed in hILC3s (Figure S2), a characteristic of tissue‐resident innate memory cells, and non‐recirculating immune cells that reside in tumor sites.^27^, ^28^ ILC1s and ILC3s express lymphoid tissue‐homing receptors but can switch the expression of homing receptors to migrate to different tissue sites.^29^ CCR6 is one such homing receptor characteristically expressed by ILC3 subsets^29^ but we found that in addition to hILC3s, subsets of hILC1s also express CCR6 (Figure S2), suggesting that certain Th17‐like hILCs may use CCR6 to migrate to bladder tissues. Collectively, these data support transcriptional and regulatory profiles of bladder tumor‐infiltrating hILCs, especially hILC1s, that differ from published hILC profiles.

### 3.3 Bladder tumor ILC1 clusters express Th17 differentiation profiles

**FIGURE 3:**
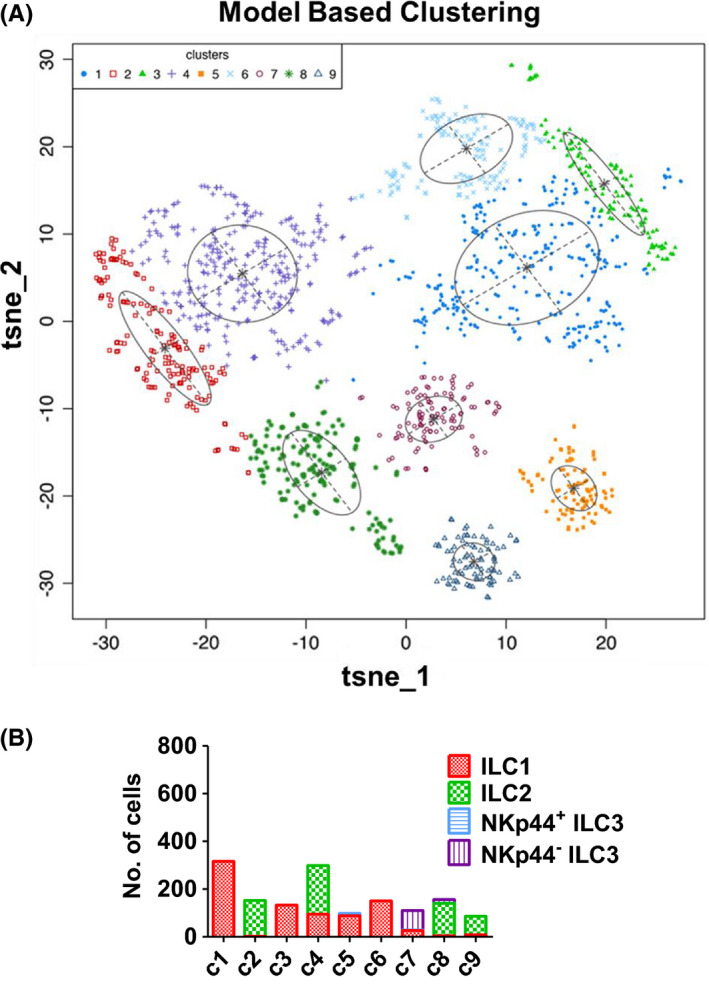
Model‐based clustering reveals nine clusters of ILCs. (A) Model‐based clustering was performed on the t‐SNE coordinates of ILCs with R package *mclust*. Model‐based clustering revealed nine unique clusters of ILCs. (B) Number of ILC1s, ILC2s, and ILC3s in the nine clusters of ILCs is plotted

We next took an agnostic approach to examine similarities between bladder hILC subgroups using model‐based clustering. Model‐based clustering identified nine clusters of bladder hILCs (Figure 3A), which were not defined by the major hILC subtypes 1–3. For example, hILC1s were present with variable frequency in each of the nine clusters (Figure 3B). hILC2s were observed in clusters 2, 4, 8, and 9. NKp44^+^ hILC3s were present in cluster 5 along with hILC1s. NKp44^−^ hILC3s were mostly observed in clusters 7 and 8 (Figure 3B). These data show that hILC1s contained certain subsets that are phenotypically similar to hILC2s and hILC3s. Also, hILC1s were identified in all nine clusters indicating that hILC1 is the most phenotypically heterogeneous group among bladder tumor hILCs. None of the nine clusters were significantly associated with the tumor stage.

**FIGURE 4:**
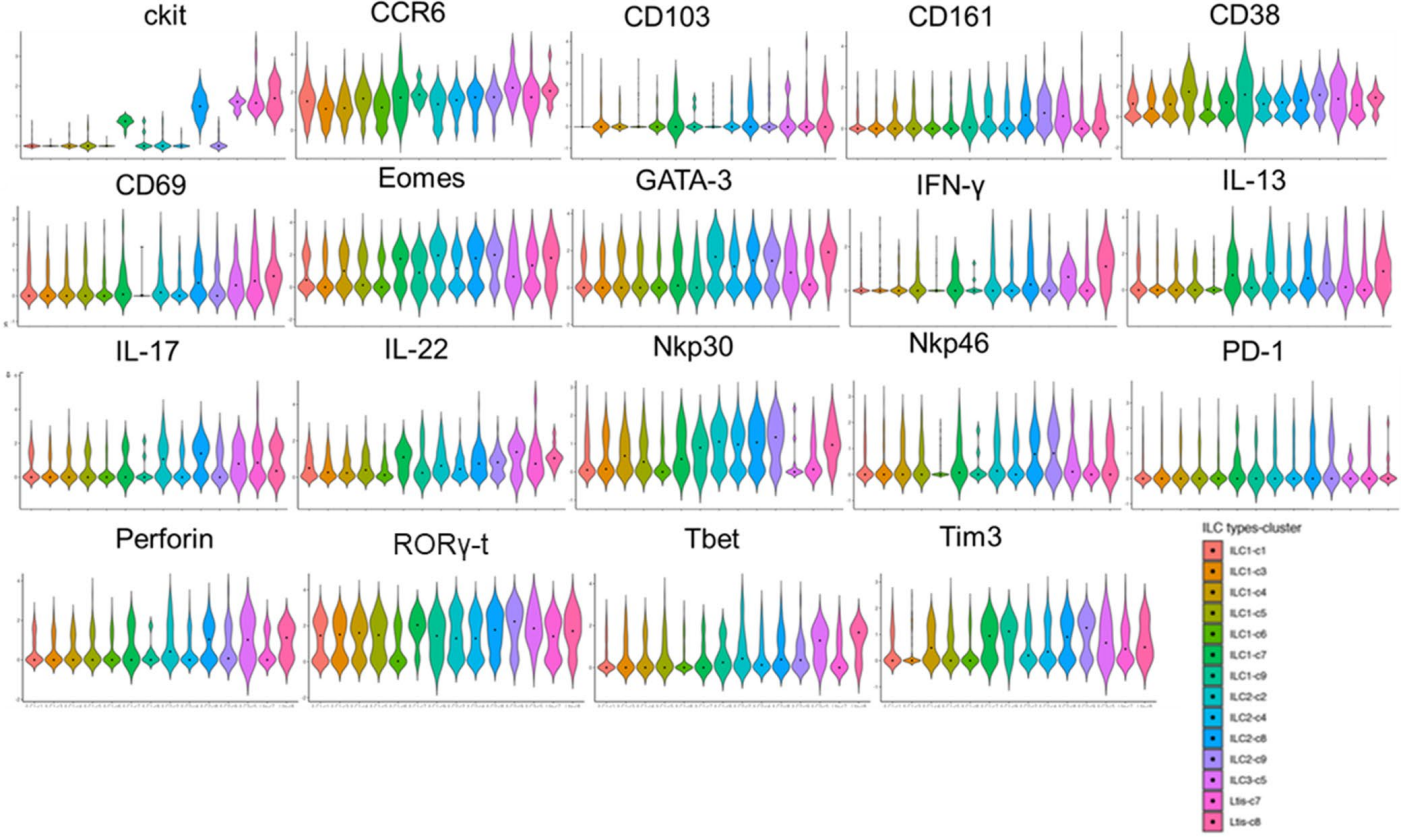
Different expression of 19 markers in the 9 clusters of ILCs. Violin plots showing the expression distribution of selected genes in the nine clusters of ILCs.

To determine the relevance of model‐based clustering to the functional activity of hILC subtypes, the nine hILC clusters were separated based on model‐based clustering and characterized by the presence of activating/inhibitory receptors, cytokines, and transcription factors (Figure 4). The expressions of Tbet, IFN‐γ, and perforin which are associated with Th1 differentiation and hILC1s, were significantly higher (*p* < 0.01) in certain subsets of hILC2s (hILC2s in clusters 2 and 8) compared with subsets of hILC1s (hILC1s in clusters 3 and 4) (Figure 4). Th17 transcription factor, RORγt, and its corresponding cytokines, IL‐17 and IL‐22, also showed high expression in several subsets of hILC1s (clusters 1, 3, 4, and 5).

Activation markers, NKp30 and NKp46 showed a significantly lower level of expression in hILC1s (cluster 1) compared with hILC2s in clusters 2 and 4. hILC1s in cluster 1 also had a significantly lower (*p* < 0.03) expression of activation marker, CD69 compared with certain hILC2s (clusters 2 and 4) and NKp44^−^hILC3s (cluster 8). Furthermore, certain clusters of hILC1s (Clusters 7 and 9) expressed high amounts of exhaustion molecules such as PD‐1 and Tim3 among other clusters (Figure 4). These data are significant because they identify novel clusters of hILCs, not defined by traditional Th differentiation profiles and support that Th17 rather than Th1 differentiation profiles are displayed by most bladder intratumoral hILC1 clusters.

## 4 DISCUSSION

hILCs play important roles in inflammation and bacterial defense at mucosal surfaces but their importance to the pathophysiology of urothelial mucosa is undefined. Here, we used high‐parametric mass cytometry to characterize the heterogeneity of muscle‐invasive bladder intratumoral non‐NK hILCs. Readouts included functional profiling of groups 1–3 ILCs and identification of nine clusters of bladder intratumoral ILCs with distinct regulatory molecules and cytokine expression. Group 1 hILCs were the most frequent non‐NK intratumoral hILCs and their presence was associated with higher tumor stage. Unexpectedly, these bladder intratumoral hILC1s did not produce high levels of IFN‐γ or Tbet, characteristic of Th1 differentiation. Instead, bladder intratumoral group 1 hILCs expressed IL‐17, IL‐22, and RORγt, indicating a Th17‐like phenotype. This supports a unique phenotype and a potential prognostic significance of intratumoral group 1 hILCs in bladder cancer.

ILCs have been described in several translational settings of human cancer, including both tumor‐promoting and tumor‐suppressive effects.^13^, ^14^, ^15^ While the range of hILCs in peripheral blood or urine of patients with cancer has been characterized,^20^, ^30^, ^31^, ^32^ less is known about intratumoral hILCs. Generally, group 1 ILCs are associated with antitumor properties, including direct cytotoxicity^13^ and high expression of antitumor cytokines including IFN‐γ, perforin, and granzyme B.^7^, ^13^, ^26^, ^32^, ^33^ Compared with normal adjacent tissue, a higher frequency of activated tumor protective hILC1s, marked by expression of high levels of CD69 and CD44,^34^ were described in gastrointestinal tumors. In late‐stage colon cancer, tumor‐infiltrating hILC1s showed lower frequencies with reduced IFN‐γ production and high levels of inhibitory receptors,^33^ suggesting that tumor protective hILC1s can become dysfunctional during cancer progression. On the other hand, IL‐17 and IL‐22 production by RORγt‐expressing Th17 T cells or Th17‐like ILCs are associated with tumor progression in several tumor models.^14^, ^35^, ^36^, ^37^, ^38^ Depletion of IL‐17^+^IL‐22^+^ colonic innate lymphoid cells prevented the development of invasive colon cancer.^14^ Other pro‐tumorigenic functions of IL‐17 include promotion of angiogenesis^39^ and recruitment of tumor‐promoting myeloid cells.^40^ We found that in addition to hILC3s, hILC1s also secreted Th17‐like cytokines and effector molecules.

The unexpected Th17‐like differentiation of group 1 hILCs in bladder tumors could be explained by functional plasticity driven by an inflammatory bladder tumor microenvironment. The effect of ILCs on cancer growth is contingent on the type of cancer, the presence of different cytokines and chemokines in the microenvironment, or the type of neighboring cells present in the tumor microenvironment.^13^, ^14^, ^16^ Chronic inflammation, as in the case of bladder carcinogenesis,^41^, ^42^ has been demonstrated to help Type 17 T‐cell development.^43^, ^44^ Tumor‐associated fibroblasts and myeloid cells in the tumor microenvironment create the cytokine milieu that fosters pro‐tumorigenic Type 17 T‐cell generation and recruitment.^45^, ^46^ Thus, the inflammatory bladder microenvironment could contribute to the conversion of group 1 ILCs to Th17‐like phenotype, analogous to the conversion of traditional tumor‐protective ILC1 cells into IL‐17‐producing ILCs in lung cancer.^47^ So, IL‐17 and IL‐22 blocking strategies including IL‐17/IL‐17R and IL‐22/IL‐22R neutralizing antibodies^48^, ^49^ and antagonists of IL‐17 and IL‐22^48^, ^50^ hold potential as therapeutic options in bladder cancer. Immune checkpoint blockade has revolutionized bladder cancer therapy.^51^ Blocking IL‐17 increased the efficacy of anti‐PD1 and anti‐PDL1 immune therapy in colorectal and breast cancers.^52^, ^53^ So, combining anti‐IL‐17––targeting Th17‐like hILC1s––with checkpoint inhibitors can be successful in bladder cancer. Furthermore, STAT3 inhibition has been shown to indirectly inhibit type 17 response.^54^ STAT3 inhibitors are effective in bladder cancer as a single agent^55^ and in combination with chemotherapeutic agents and oncolytic virotherapy^56^; future studies can investigate whether STAT 3 inhibitors target IL‐17‐producing hILC1s in bladder cancer. The use of transgenic murine models such as IL‐22, IL‐17, and RORγt‐deficient mice^57^, ^58^, ^59^ will help to delineate the role of Th17‐like hILC1s in bladder cancer. IL‐22 and RORγt reporter mice^60^, ^61^, ^62^ can also be useful in identifying and isolating IL‐22 and RORγT‐expressing hILC1s. RORγt^+^ hILC1s can be targeted by Cre expression induced by Tbx21 (coding for T‐bet) with the floxed allele of RORγt,^63^ thereby preventing the effects of RORγt deficiency in non‐ILC immune cells.

Limitations of this study are noted. Based on their phenotype and association with higher stage tumors, bladder intratumoral group 1 ILCs are predicted to be associated with decreased survival, but the cohort size limits the ability to assess the association of intratumoral hILCs with survival outcomes. Further work is also needed to test the function of these intratumoral hILCs directly, including cellular cytotoxicity, memory, and exhaustion to validate phenotypes defined by cytometric analysis. Published identification strategies of hILCs are variable and existing gating strategies are limited in ability to detect extremely rare populations of intratumoral hILCs. A total of 5,000 CD45^+^ lymphocytes were sampled from each clinical tumor sample to minimize biases from sampling variable numbers of cells in each sample. As a result, some rare ILCs were likely not identified in our analysis and these could contain more unique subtypes of hILCs. Nevertheless, we used a well‐established and comprehensive panel for the identification of hILCs.^21^ Finally, our panel did not include markers for effectively identifying lymphoid tissue inducer cells (LTis) (NKp46^−^Ox40L^+^CD30L^+^lymphotoxin‐α^+^ ILC3s)^64^ but LTis are detected mainly in fetal lymphoid tissue^65^ and adult secondary lymphoid organs^64^ and are less likely to be present in bladder tumors.

## 5 CONCLUSION

Group 1 hILCs are the most prevalent non‐NK hILCs among bladder tumor‐infiltrating lymphocytes. These intratumoral group 1 ILCs are associated with higher bladder tumor stage and exhibit a Th17‐like differentiation phenotype, marked by high expression of RORγt, IL‐17, and IL‐22 and low expression of Th1 and Th2 transcription factors and cytokines. These observations identify group 1 non‐NK hILC1s as potentially important determinants of bladder cancer progression. Future studies are needed to elucidate the biological function and effector mechanisms of these Th17‐like group 1 hILCs. Cre‐inducible knockdown of RORγt specifically in Tbet‐expressing hILC1s can be useful in delineating the functional relevance of Th17‐like hILC1s in bladder cancer. Antagonistic inhibitors of IL‐17 and IL‐22 need to be investigated in future studies to target Th17‐like hILC1s as novel treatment strategies in bladder cancer.

## CONFLICT OF INTEREST

The authors have declared that they have no conflict of interest.

## ETHICAL APPROVAL

Patients were recruited through a local Institutional Review Board (IRB)‐approved observational cohort study, which collected clinical data and bladder tissue for analysis (IRB # BCR20120159H). We obtained IRB approval from UTHSA to conduct minimal risk human research. We used the IRB‐approved consent form to obtain written consent from all participants and all identifying information was removed in order to maintain participants’ right to privacy. Through initial and ongoing approval of our research, we ensured we were complying with all relevant federal and institutional regulations and policies related to the protection of human subjects. This study's involvement with human subjects complies with the Declaration of Helsinki.

## Supporting information

Supplementary Material

## Data Availability

Raw data were generated at [Bioanalytics and Single‐Cell Core]. Derived data supporting the findings of this study are available from the corresponding author [RSS] upon request.
