# Supplementary material for: Bladder tumor ILC1s undergo Th17‐like differentiation in human bladder cancer
Source: Cancer Med. 2021 Sep 8;10(20):7101–10. doi: 10.1002/cam4.4243 (PMC8525153; doi:10.1002/cam4.4243)
Supplement: Supplementary file 1 — Supplementary Material [file CAM4-10-7101-s001.docx]

**Supplementary Tables**

**
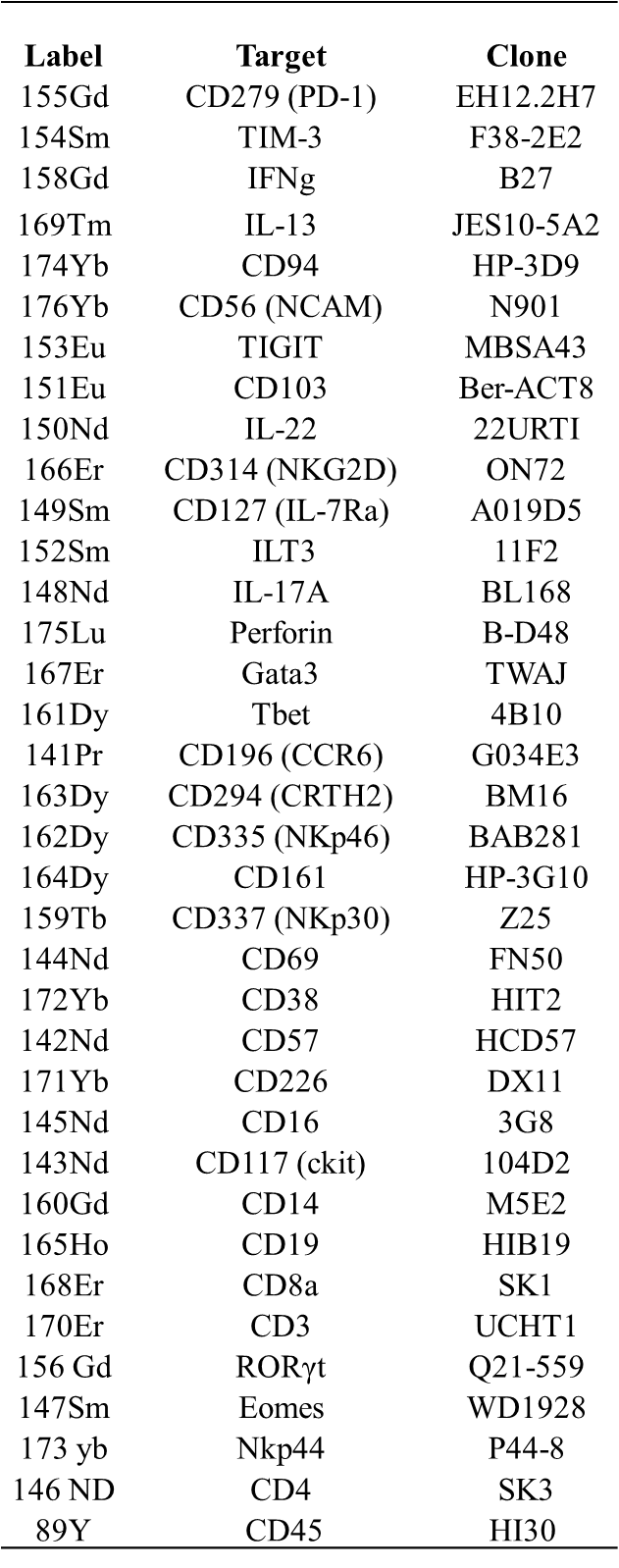
**

**Supplementary Table 1. Clones and metal labels of antibodies (CyTOF).** Clones and metal labels of antibodies used in the CyTOF analysis are listed.

**Difference of mean expression of characteristic transcription factors and cytokines between ILC types**


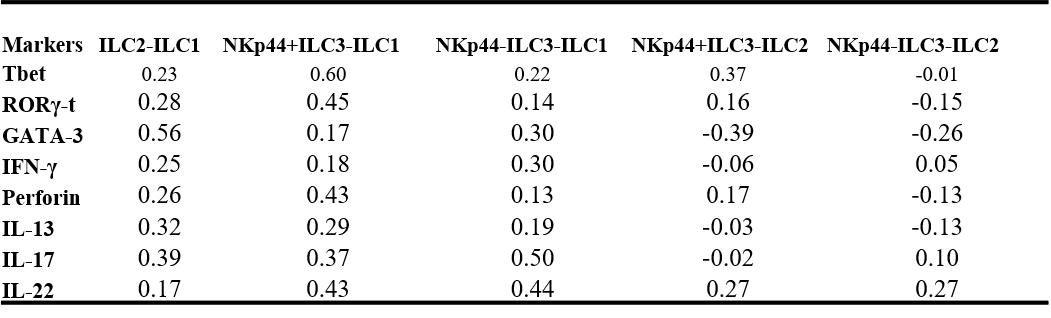


**p values of multiple comparison**


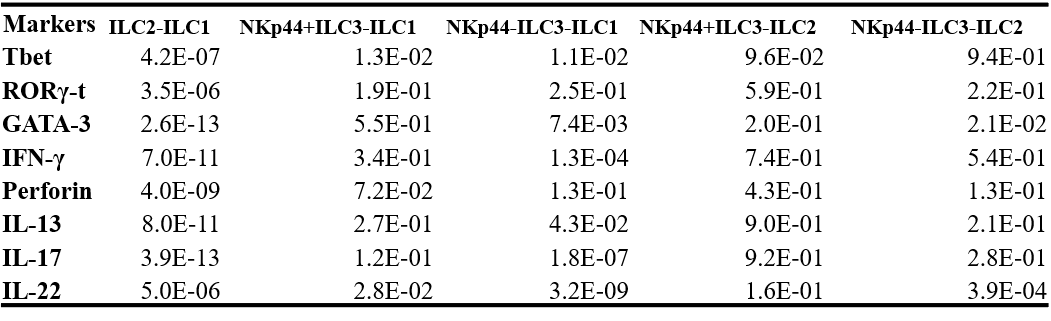


**Supplementary Table 2. Multiple comparison of characteristic transcription factors and cytokines between ILC types.** Difference of mean expression of characteristic transcription factors and cytokines between ILCs1/2/3 and the corresponding p values are listed.

**Difference of mean expression of activation and exhaustion markers between ILC types**


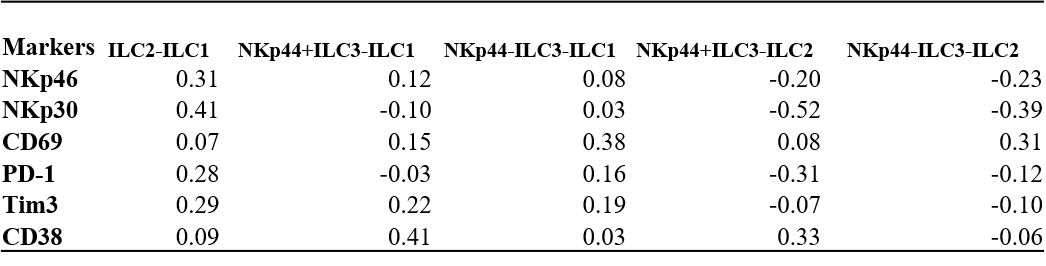


**p values of multiple comparison**


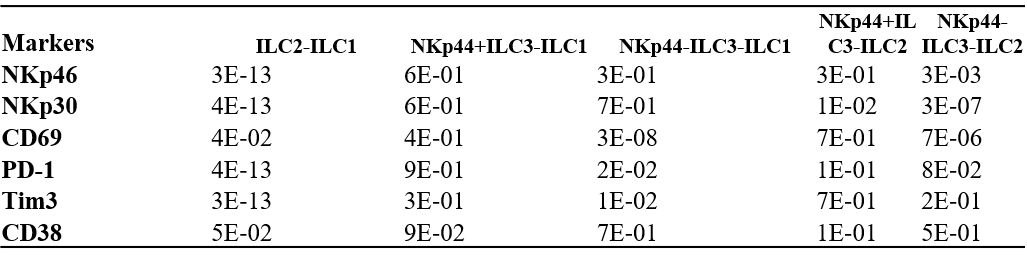


**Supplementary Table 3. Multiple comparisons of activation and exhaustion markers between ILC types.** Difference of mean expression of activation and exhaustion markers between ILCs1/2/3 and the corresponding p values are listed.

**Supplementary Figures**

**Figure Legends**

**Supplementary Figure 1. Different ILCs exhibit characteristic cytokine and regulatory profile but differ in their activation and exhaustion phenotype.** Human bladder tumor tissues (n=21) was harvested and processed into single cell suspensions and analyzed with CyTOF. (**A**) Subsets of ILC1/2/3 expressing the indicated markers are calculated as percentage of ILC1s ILC2s and ILC3s. (**B**). Percentages of ILC1,2,3 expressing activation receptors (NKp46, NKp30, CD69) and suppressive markers (CD38, PD-1, Tim-3) are plotted.

**Supplementary Figure 2. Different ILCs exhibit differential expression of activation receptors and immune suppressive molecules.** Different expression of activation receptors and immune suppressive markers in ILC1, ILC2, ILC3s are plotted as violin plots.


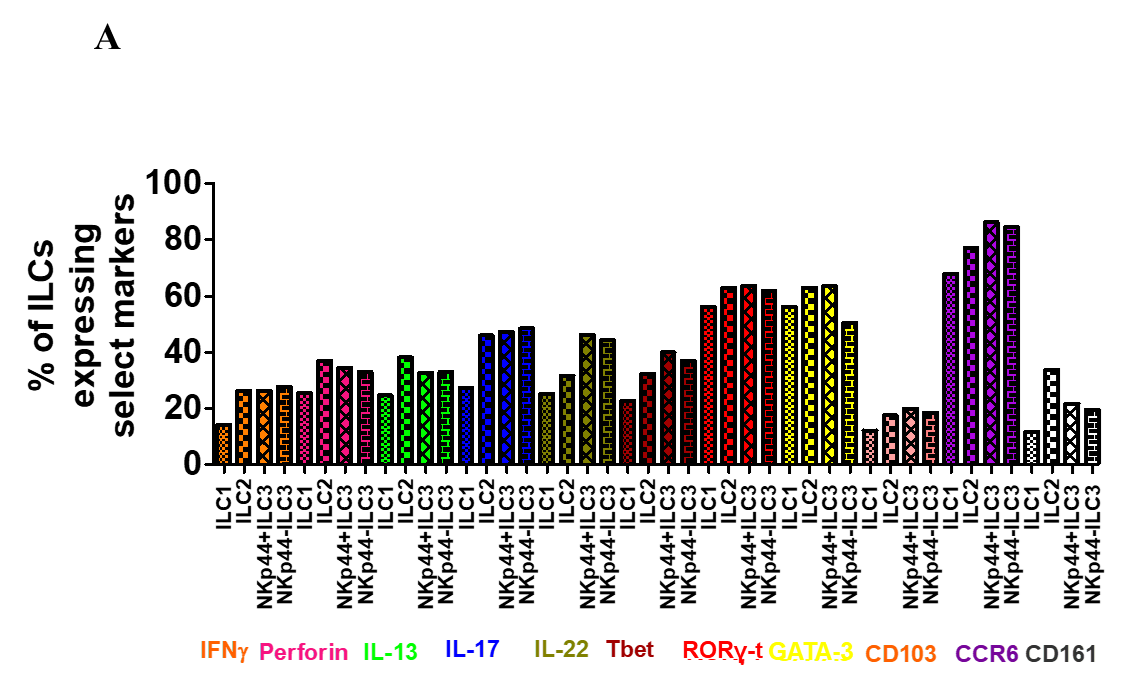


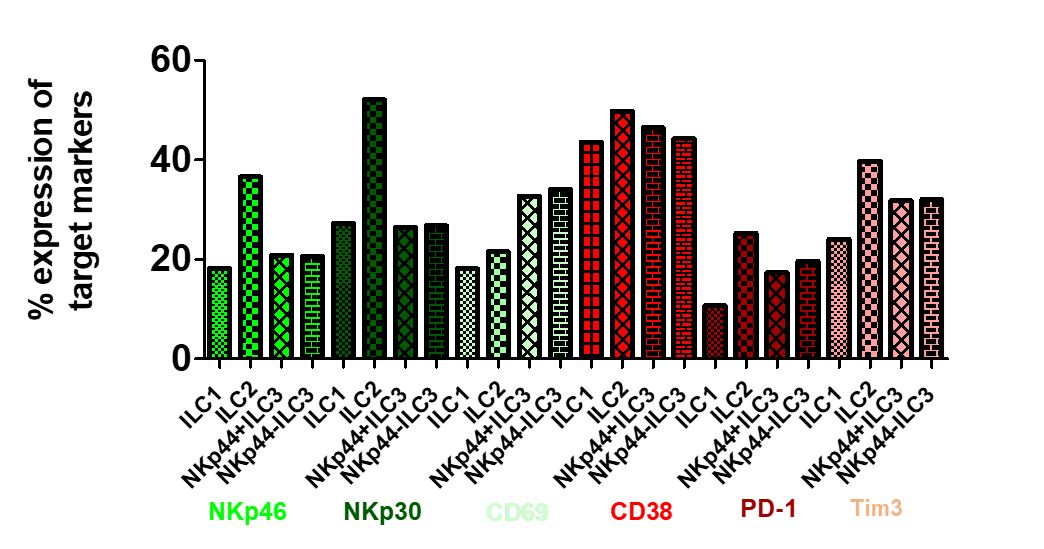


**B**

**Supplementary Figure 1. Different ILCs exhibit characteristic cytokine and regulatory profile but differ in their activation and exhaustion phenotype**


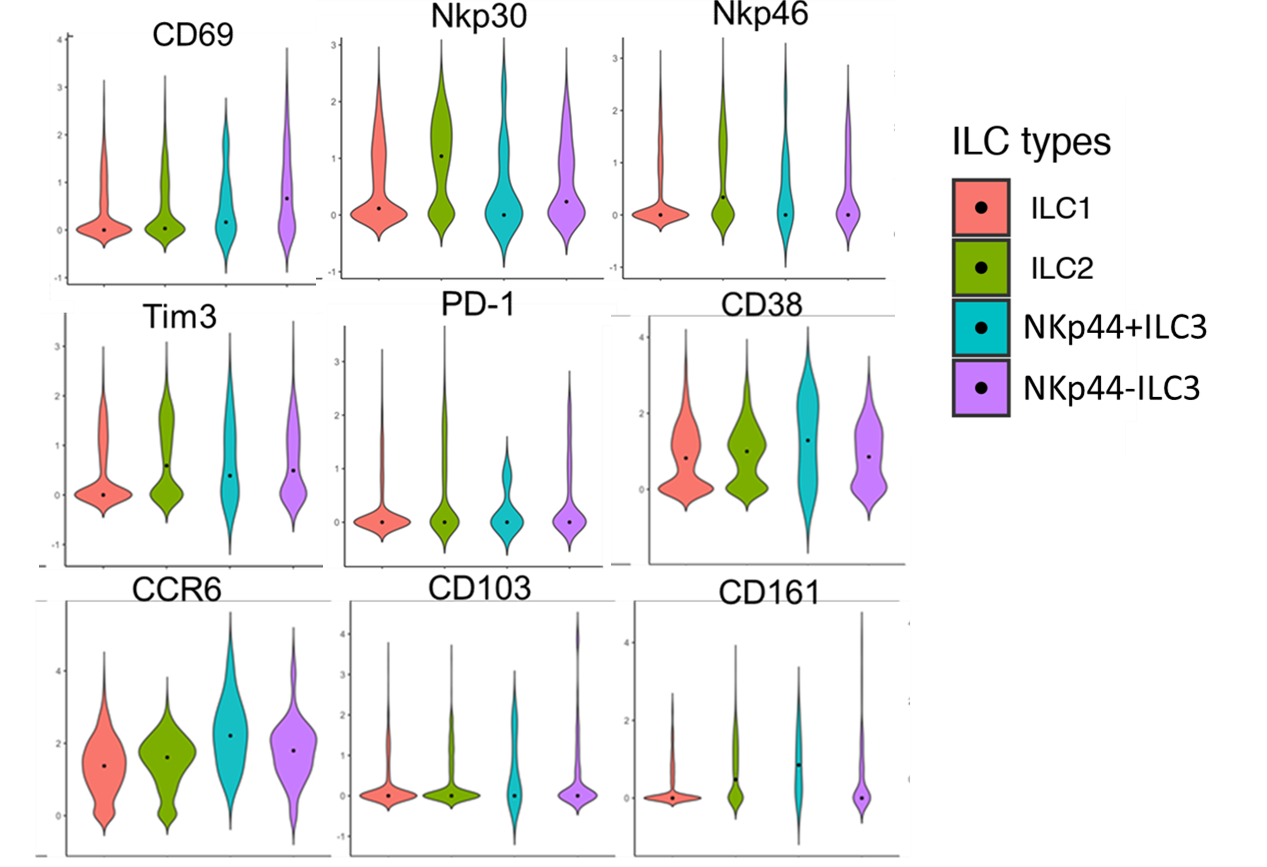


**Supplementary Figure 2. Different ILCs exhibit differential expression of activation receptors and immune suppressive molecules.**
